# Estrogen enhances the proliferation and migration of ovarian cancer cells by activating transient receptor potential channel C3

**DOI:** 10.1186/s13048-020-00621-y

**Published:** 2020-02-22

**Authors:** Shengnan Li, Kuo Jiang, Jia Li, Xiaohua Hao, Wenguang Chu, Ceng Luo, Yuanyuan Zhu, Rougang Xie, Biliang Chen

**Affiliations:** 1 Department of Gynecology and Obstetrics, Xijing Hospital, Air Force Medical University, Xi’an, 710032 Shannxi China; 2 Department of Spine Surgery, Honghui Hospital, Xi’an Jiaotong University College of Medicine, Xi’an, 710054 Shaanxi China; 3 Department of neurobiology, Air Force Medical University, Xi’an, 710032 Shannxi China

**Keywords:** Epithelial ovarian cancer, Estrogen, Transient receptor potential channel C3 (TRPC3), Cell proliferation,cell migraiton

## Abstract

**Background:**

Recent studies have suggested that estrogen (E2) plays an important role in epithelial ovarian cancer (EOC). However, the mechanism of E2 in ovarian cancers is unclear. The purpose of this study was to investigate the effect of E2 on ovarian cancers and illuminate the mechanism of E2 in promote ovarian cancers proliferation.

**Results:**

We demonstrated that E2 stimulated the proliferation and invasion of ovarian cancer cells. In this study, ovarian cancer specimens were also analyzed for transient receptor potential channel C3 (TRPC3) expression; TRPC3 expression levels were higher in ovarian cancer samples than in normal ovarian tissue samples. Previous studies have shown that TRPC3 contributes to the progression of human ovarian cancer. In this study, we further investigated the interaction between E2 and TRPC3. We found that E2 stimulation enhanced the expression of TRPC3 at both the mRNA and protein levels. E2 stimulation enhanced the influx of Ca2^+^. Moreover, siRNA-mediated silencing of TRPC3 expression inhibited the ability of E2 to stimulate the influx of Ca2^+^.

**Conclusions:**

In conclusion, TRPC3 plays a significant role in the stimulatory activity of E2 and could be a therapeutic target for the treatment of EOC. Furthermore, this study elucidates the molecular mechanism by which E2 promotes the proliferation and migration of EOC cells.

## Introduction

Epithelial ovarian cancer (EOC) is the most common type of gynecological cancer. Annually, 230,000 women worldwide will be diagnosed with EOC, and 150,000 will die [1]. EOC represents the seventh most commonly diagnosed cancer among women globally and has a 46% 5-year survival rate after diagnosis. One of the main factors contributing to the high death-to-incidence ratio is the advanced stage of disease at the time of diagnosis [2]. The cause of this disease is not clear. Epidemiological investigation has shown that EOC is influenced by hormones, reproduction, genetics, inflammation, life habits and so on. The main treatments include surgery and systemic therapy [3–5]. However, the pathogenesis of EOC remains unclear. To date, numerous hypotheses have been proposed to explain the etiology of ovarian cancer [6–8]. Thus, new insights into the cause of EOC are urgently needed.

The ovaries are the main source of female reproductive steroid hormones. Several lines of evidence support the view that the growth of many ovarian cancers is regulated by estrogen (E2, [9, 10]). Moreover, E2 is one of the most important hormones in the female reproductive system and is mainly secreted by the ovaries. It exhibits a broad spectrum of physiological functions ranging from regulation of the menstrual cycle and reproduction to modulation of bone density, brain function, and cholesterol mobilization [11–13]. Both of the major isoforms of estrogen receptor (ER), ER-alpha and ER-beta, are expressed in ovarian cancers [14]. However, the mechanism involving ER in ovarian cancers is unclear.

Ca^2+^ is a second messenger that plays a major role in the regulation of cellular functions such as secretion, cell growth and death, and contraction [15, 16]. The canonical transient receptor potential channels (TRPCs), a family of nonselective cation channels mainly used by Ca^2+^, can be involved in calcium influx and downstream pathways, regulating cell survival, proliferation and carcinogenesis via intracellular translocation induced by hormones and growth factors [17–19]. TRPCs are ubiquitously distributed in the body and play essential roles in human physiology and pathophysiology. TRPC3 protein levels are markedly increased in human ovarian cancer specimens compared to normal ovarian tissue specimens, and decreasing the expression of TRPC3 reduces the proliferation of cultured human ovarian cancer cells [20].

Here, we aimed to identify the expression of TRPC3 in EOC tissue samples and ES2, SKOV3, OVCAR, and HEY cells treated with E2 to determine the roles of TRPC3 in the E2-mediated regulation of EOC-related cell proliferation using TRPC3-specific siRNA interference. In addition, we compared the expression of TRPC3 genes in human ovarian cancer tissue samples from patients with that in normal ovarian tissue samples.

## Materials and methods

### Human tissue samples

Normal ovarian tissue comes from 6 patients with benign uterine lesions and normal ovaries who has had a hysterectomy and has had the ovarian tissue removed. Seven ovarian cancer patient tissue samples were obtained from the gynecology and obstetrics department of Xijing Hospital (Xi’an, China). Prior to the experiment, all patients were informed of the purpose of the study as well as the procedures and voluntarily agreed to provide tissue. Written consent was obtained from all participants, and all protocols were approved by the Ethics Committee of Xijing Hospital, which is affiliated with the Air Force Medical University.

### Cell culture

ES2 and SKOV3 cells were cultivated in McCoy’s 5A medium (HyClone/high glucose, GE Healthcare life sciences, Logan, Utah),and OVCAR and HEY cells were cultured in 1640 medium supplemented with 10% fetal calf serum (FBS; BI) and 2 mM L-glutamine at 37 °C and 5% CO_2._ All cells were purchased from GENE (Shanghai, China). E2 was purchased from Sigma-Aldrich (St. Louis, Missouri,USA). E2 was dissolved into DMSO. FBS was treated with activated carbon when added during E2 stimulation, and the medium used was phenol-free red medium. Cells were treated with 10 nM, 100 nM or 1 μM E2 for 24 h, 48 h or 72 h. DMSO was used as a negative control.

### Cell vitality assay

In total, 5 × 10^3^ cells were grown in a 96-well plate with or without different concentrations of E2 (10 nM, 100 nM, and 1 μM). DMSO was used as a negative control. After incubation for 24 h, 48 h or 72 h, 20 μL of 5 mg/ml 3-(4, 5)-dimethylthiahiazo-3, 5-di-phenytetrazoliumromide (MTT) was added to each well. The cells were incubated for 4 h at 37 °C, and then the culture medium was replaced with 150 μL of DMSO (Sigma). The absorbance of the formazan product at 490 nm was measured with a plate reader. All experiments were performed with three replicates.

### Transwell migration assay

SKOV3 cell migration was examined with chemotaxis assays. SKOV3 cells were starved for 12 h in McCoy’s 5A medium without FBS. Then, the cells were seeded (2 × 10^4^ cells) in the upper compartment of a Transwell plate (24-well plates, the poor size of Transwell is 3 μM) with 1 μM E2. McCoy’s 5A medium supplemented with 10% FBS and 1 μM E2 was added to the bottom of the well to serve as a chemoattractant for the SKOV3 cells. For the control group, an equal volume of DMSO instead of 1 μM E2 was added to the medium. Next, the cells were allowed to migrate for 24 h, after which the cells were fixed, stained with crystal violet and quantified by ImageJ software. For each biological replicate, at least three random fields were imaged. The influence of E2 on SKOV3 cells transfected with TRPC3 SiRNA was also investigated by chemotaxis assays. For this experiment, cells were allowed to migrate for 48 h.

### siRNA transfection

A TRPC3-specific siRNA and Sicon were purchased from Shanghai GenePharma Co., Ltd. The TRPC3-specific siRNA and Sicon were transfected into SKOV3 cells using Lipofectamine 2000 (Invitrogen, State of California,USA). The wells without siRNA (no siRNA) or with a scrambled siRNA or a nonspecific pool of siRNAs were set as negative controls in parallel.

#### Wound healing assay

SKOV3 and HEY cells transfected with or without TRPC3 SiRNA were cultured on 6-well plates (10^5^ cells/well) in McCoy’s 5A medium and 1640 medium. After reaching confluency, cells were starved with serum-free medium for 12 h. Then the cells monolayer was scratched horizontally with a yellow pipette tip to obtain a monolayer culture with a space without cells. Cellular debris was removed by gently washing twice with PBS and then cultured with or without 100 nM E2. Three randomly selected fields along the scraped line were photographed using a phase contrast inverted microscope. After incubation for 12, 24, 36, 48, 72 h, images were acquired and cell migration distances were evaluated by ImageJ.

#### Cell counting Kit-8(CCK-8) assay

Cell proliferation was determined by CCK-8 assay kit (MA0218-Apr-25E, Meilunbi0, China). One thousand SKOV3 and HEY cells transfected with or without TRPC3 SiRNA were cultured on 96-well plates and 100 nM E2 was added to the culture solution and cultured for 24 h, then 10 μL CCK-8 reagent was added to medium and incubated for 1 h. OD (optical density) at 450 nm was read with a microplate reader. Each experiment was performed three times independently.

### Immunocytofluorescence analysis

For immunocytofluorescence analysis, cells were fixed with 4% paraformaldehyde. Then, the cells were permeabilized with Triton X-100 and blocked with BSA. The cells were incubated with an anti-TRPC3 antibody (Alomone, ACC-016, Jerusalem, Israel) at 4 °C overnight and with a CY3–555-conjugated IgG antibody at 37 °C for 2 h in the dark on the following day. DAPI was used for nuclear staining. Images were captured with an FSX100 microscope (Olympus, Tokyo, Japan). Tissue samples obtained from patients were fixed in 4% paraformaldehyde and serially cut into sections of 16-μm thickness using a cryostat. For immunocytofluorescence, the frozen sections were subjected to antigen retrieval. An anti-TRPC3 antibody was employed as the primary antibody at a dilution of 1:200; and a cyanidin-3-labeled goat anti-rabbit secondary antibody was used to visualize TRPC3 expression. All stained slides were scanned using a confocal laser scanning microscope, and images were analyzed using ImageJ software (National Institutes of Health, MD, USA).

### Quantitative RT-PCR for TRPC3 expression

OVCAR, ES2, HEY and SKOV3 cells were seeded in 6-well dishes. After 48 h, the cells were treated with E2. Total RNA was extracted from the different cells using RNAiso Plus (Takara, Dalian, China) and was subsequently reverse transcribed into cDNA using PrimeScript™ RT.

Master Mix with random primers (Takara, Dalian, China) was used according to the manufacturer’s protocol. The expression of different genes was analyzed using SYBR® PremixEx Taq™ II and the Bio-Rad CFX System. For real-time PCR, the reaction mixtures contained 2 μL of cDNA, 0.8 μL of each primer (10 mmol^− 1^), 10 μL of SYBR green PCR Master Mix, and 0.2 μL of ROX Reference Dye II, and distilled water was added to reach a final reaction volume of 20 μL. Taq DNA polymerase was activated at 95 °C for 10 min, followed by 40 cycles of 95 °C for 15 s, 60 °C for 30 s, and 72 °C for 30 s. Quantitative RT-PCR data were normalized to the expression of the housekeeping gene GAPDH using the 2^-ΔCt^ method. The primers used in this study are shown in Table 1.

**Table 1:** Primers used in this study

Genes	Sequences (5′-3′)
GAPDH	F: ATGGGGAAGGTGAAGGTCG R: GGGGTCATTGATGGCAACAATA
TRPC3	F: CATTCCTGGCCATTGGCTACT R: GCAGACCCAGGAAGATGATGAA

### Western blot analysis

Cell lysates were extracted with a lysis buffer. Western blotting was performed with 30 μg of total protein after total protein concentrations were estimated by a BCA assay, and then the protein samples were mixed with 5X SDS-PAGE protein loading buffer (Boster, Wuhan, P. R. China) and boiled. The samples were transferred to a PVDF membrane (Millipore, Darmstadt, Germany) by using the wet transfer method after being separated on 10% SDS-PAGE gels (Boster, Wuhan, P. R. China). After blocking with 5% milk in TBST for 2 h, the membranes were incubated with an anti-TRPC3 (Alomone, ACC-016, Jerusalem, Israel) or anti-β-actin (CST, PA5–85271, Massachusetts, USA) antibody overnight at 4 °C. To verify the specificity, the membranes were incubated with control peptide antigen and the primary antibody. β-actin was used as an internal control. The membranes were incubated with a horseradish peroxidase-conjugated anti-rabbit antibody at room temperature for 1 h after washing with TBST. The blots were visualized by ECL Western blotting substrate (Millipore, Darmstadt, Germany) according to the manufacturer’s instructions.

### Measurement of intracellular Ca^2+^ signaling

SKOV3 and HEY cells were grown on coverslips and loaded with the calcium indicator Fura-2 AM (5 μM, Invitrogen) for 30 min in McCoy’s 5A medium at room temperature and away from light, washed with normal calcium desklop fluid (130 mM NaCl, 5 mM KCL, 1.2 mM MgCl_2_, 1.8 mM CaCl_2_, 10 mM HEPES, 10 mM glucose, and NaOH added to achieve a pH of 7.4) three times, continuously perfused throughout the experiment at room temperature with a nominal-calcium indicator loading buffer and challenged with ATP and E2. To investigate the effect of E2 on the expression of TRPC3 in ovarian cancer cells, SiTRPC3 and Sicon were transfected into SKOV3 and HEY cells, which were then incubated on a glass coverslip for 48 h. Then, 1 μM E2 was added to the medium at the start of detecting intracellular Ca2^+^. Excitation light was supplied via a Polychrome II polychromator (TILL Photomics, Oberhausen, Germany), and emission was detected by a Sensicam CCD camera (PCO Computer Optics, Kelheim, Germany). Ca^2+^-sensitive Fura-2 AM fluorescence was measured ratiometrically at 340/380-nm wavelengths with an emission wavelength of 510 nm. Recordings were analyzed by using Axon Imaging Workbench (Axon Instruments, Ismaning, Germany).

### Statistical analysis

Each experiment was repeated at least three times, and the data are presented as the mean ± SEM. Kaplan-Meier survival was analyzed by the log-rank test, and other comparisons were evaluated with one-way ANOVA. All data were graphed and analyzed statistically using GraphPad Prism 5 (GraphPad software, Inc.) and SPSS20 (IBM, Inc.). A *P*-value < 0.05 was considered to indicate a statistically significant difference.

## Results

### E2 promoted the proliferation and migration of different ovarian cancer cells

**Fig. 1:**
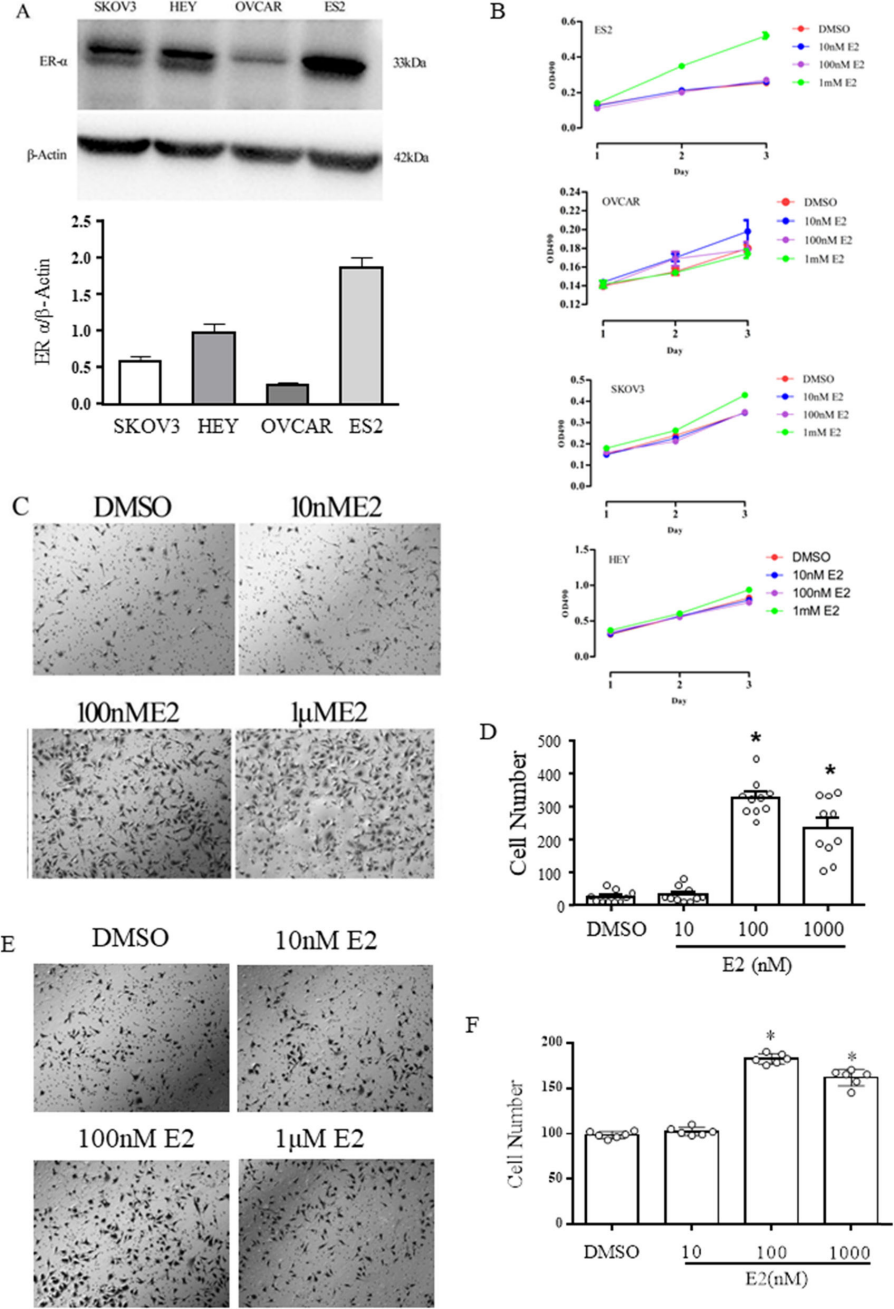
E2 increased proliferation and migration in ovarian cancer cells. **a** The expression levels of ER-α in ovarian cancer cells (SKOV3, HEY and ES2 cells) were detected by Western blot analysis. β-Actin was used as a loading control. **b** The ovarian cancer cell lines ES2, OVCAR, SKOV3, and HEY were treated with 10 nM, 100 nM or 1 μM E2 for different times (24 h, 48 h or 72 h). Cell growth was detected by an MTT assay (OD490 nm). The ovarian cancer cell line SKOV3((**c**) and (**d**)) and HEY ((**e**) and (**f**))was treated with 10 nM, 100 nM or 1 μM E2 for 48 h. DMSO was used as a negative control. Cell migration was analyzed by a Transwell migration assay. The experiment was repeated three times

**Fig. 2:**
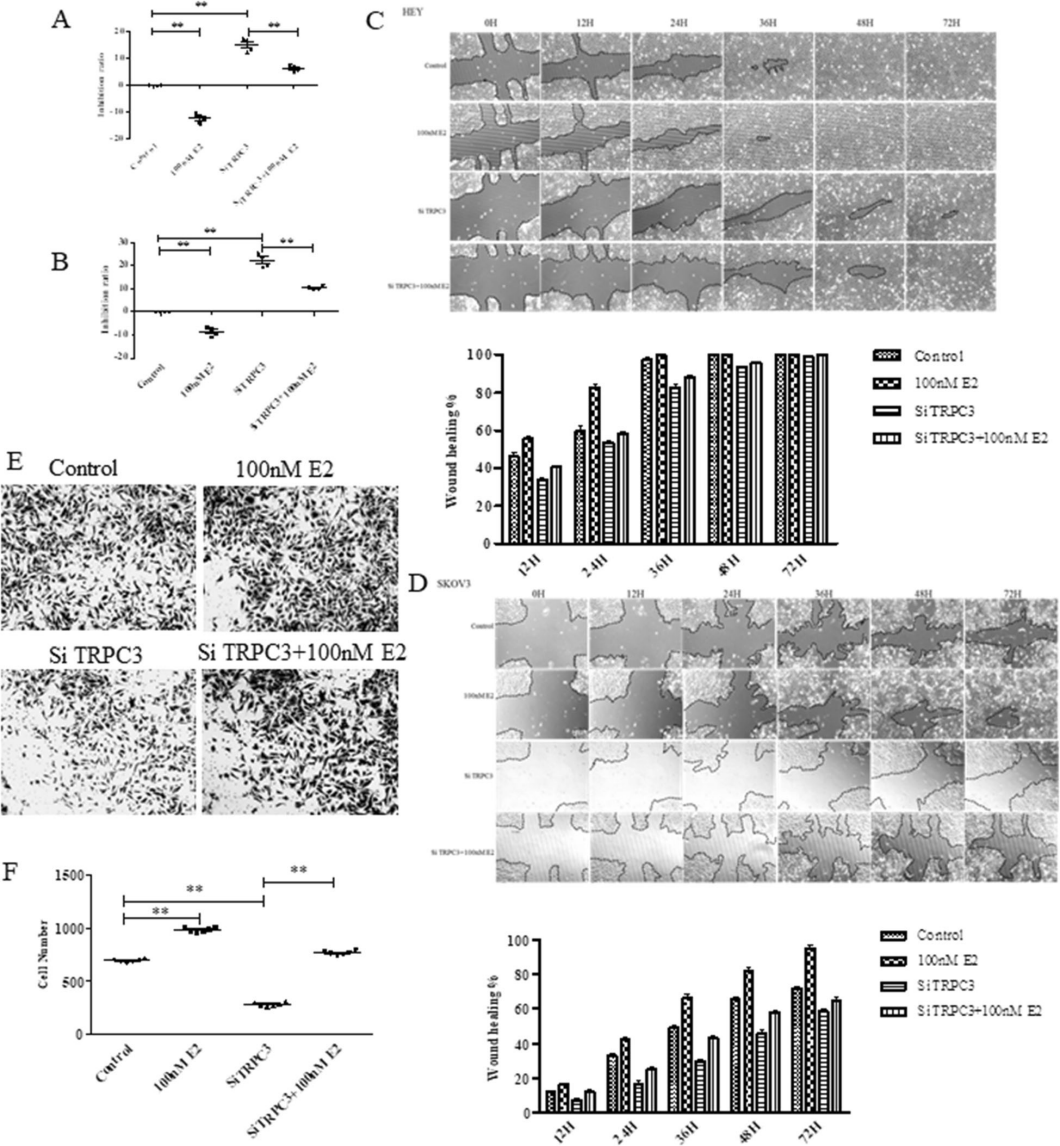
Knocking down TRPC3 expression influenced the cell behaviors. The effect of TRPC3 and E2 on cell proliferation was detected by CCK8 assay (**a** and **b**) and wound healing assay (**c** and **d**). **a c** HEY cells and **b d**SKOV3 cells. **e**, **f** SKOV3 cell was transferred with Si TRPC3 treated with or without 100 nM E2. Cell migration was analyzed by a Transwell migration assay. The experiment was repeated three times

To investigate the function of ER-α in ovarian cancer, we detected the expression of ER-α in different ovarian cancer cell lines. The cell line ES2 had the highest expression level among the four ovarian cancer cell lines, while the cell line OVCAR had the lowest expression level (Fig.1a). Next, we detect the cell viability of four cell lines under E2 conditions. Different cell lines were grown with or without different doses of E2. Cell viability was measured by an MTT assay. The cell viability of all cells, especially that of ES2 cells, was influenced by 1 μM E2 (Fig. 1b). To further understand the influence of E2 on cell migration, in vitro Transwell assays was performed. The Transwell assays showed that SKOV3 and HEY cell migration was significantly increased under E2 conditions (Fig. 1c and d and Fig.1e and f). The cell number decreased when SKOV3 and HEY cells transferred with Si TRPC3 while E2 can increase the cell number (Fig. 2e and f). CCK8 assay and wound healing assay results shown that E2 promote the cell proliferation while silenced of TRPC3 inhibit the cell proliferation which can reverse by E2 in HEY and SKOV3 cells (Fig. 2). All results demonstrate that E2 enhances cell proliferation and migration.

### Tissue samples from ovarian cancer patients showed high expression of TRPC3

**Fig. 3:**
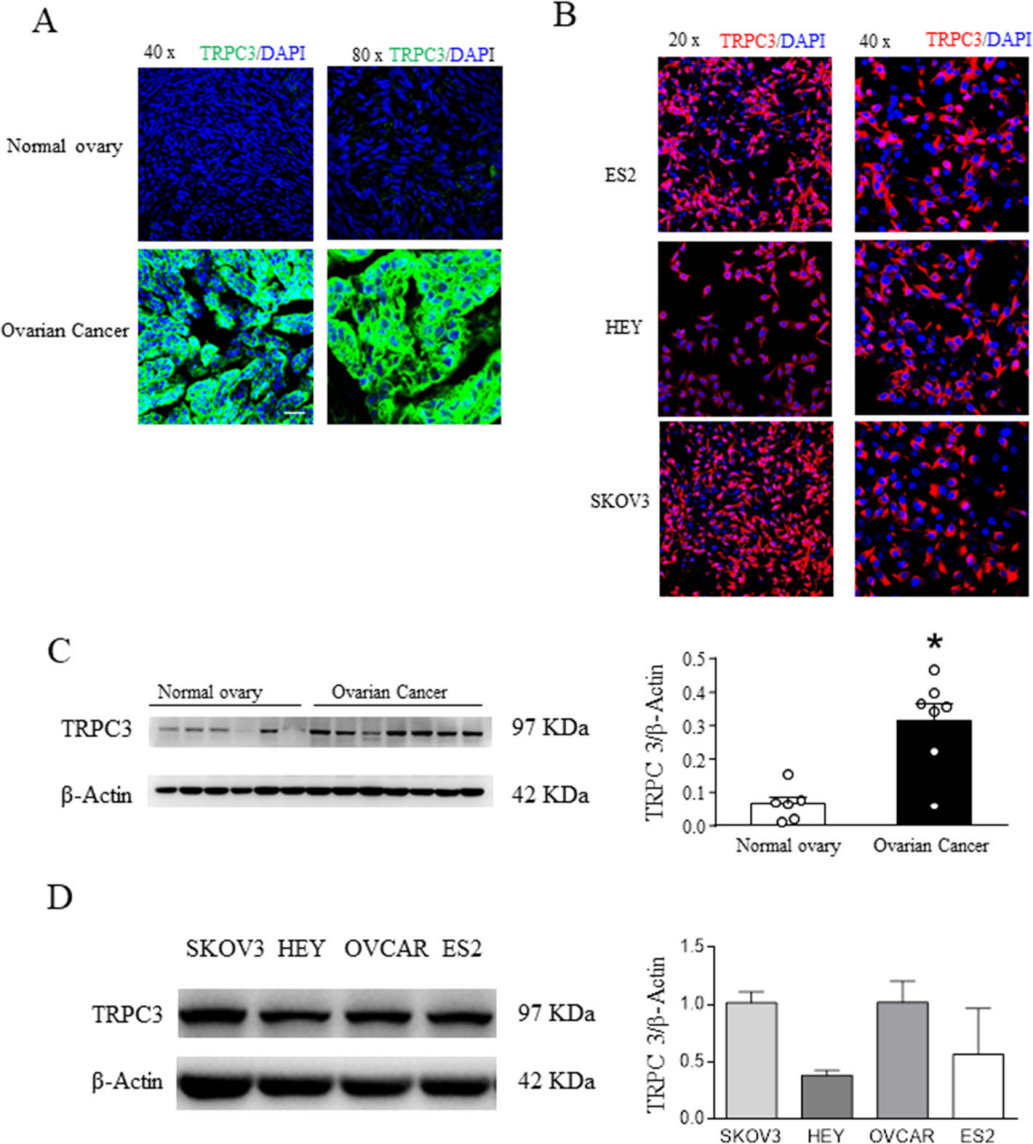
TRPC3 expression in ovarian cancer cells and tissues. **a** The expression levels of TRPC3 in ovarian cancer tissue samples and ovarian tissue samples from patients with benign uterine lesions and normal ovaries who has had a hysterectomy and has had the ovarian tissue removed detected using immunofluorescence. **b** The expression levels of TRPC3 in ovarian cancer cells (SKOV3, HEY and ES2 cells) were detected using immunofluorescence. The TRPC3 protein was immunofluorescently labeled and imaged using confocal microscopy. DAPI was used as a nuclear staining marker. **c** The expression levels of TRPC3 in ovarian cancer tissue samples and ovarian tissue samples from benign lesion patients detected using Western blot analysis. **d** Western blot analysis was used to detect TRPC3 expression in four ovarian cancer cell lines. β-Actin was used as a loading control

To detect the expression of TRPC3 in ovarian cancer tissue samples, ovarian tissue samples from ovarian cancer and uterine fibroid patients were collected to analyze the expression of TRPC3. The ovarian tissue samples from the patients with ovarian cancer showed higher protein expression of TRPC3 (Fig. 3c) than the ovarian tissue samples from the uterine fibroid patients. Immunocytofluorescence analysis showed that the expression of TRPC3 in serous ovarian cancer tissue was higher than that in normal tissue (Fig. 3). We also used blocking peptide to examine the specificity of the band around 97 kDa. Result shown that the band around 97 kDa specifically represent TRPC3(Fig S2).

### Expression levels of TRPC3 differed among ovarian cancer cells

To investigate whether E2 promotes the proliferation of ovarian cancer cells through TRPC3, we detected the expression of TRPC3 in different ovarian cancer cell lines through Western blot and immunocytofluorescence analyses. The expression of TRPC3 in SKOV3 and OVCAR cells was higher than that in HEY and ES2 cells (Fig. 3b and d).

### E2 increased the expression of TRPC3 in different ovarian cancer cells

**Fig. 4:**
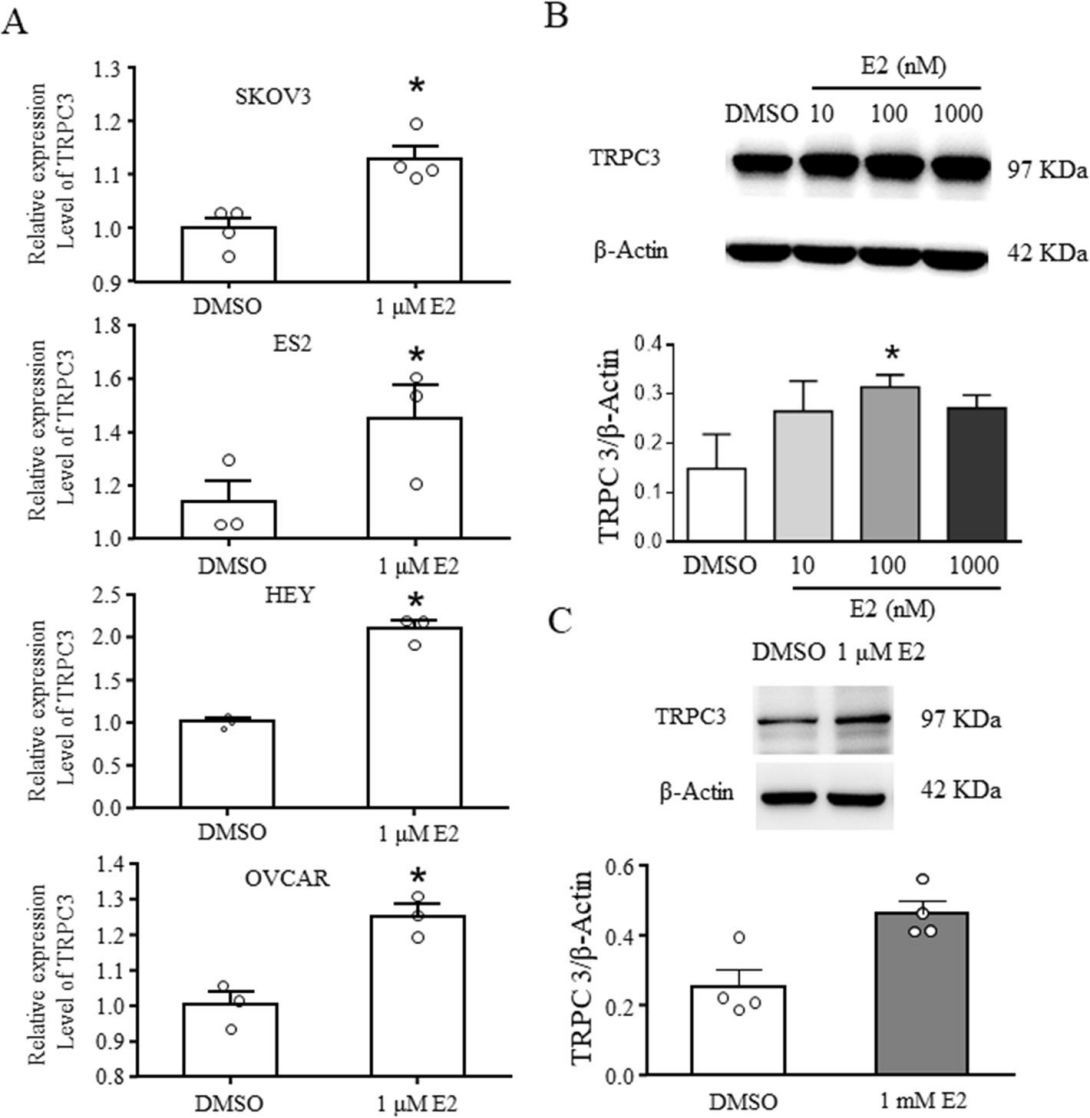
E2 increased the expression of TRPC3 in ovarian cancer cells. **a** The ovarian cancer cell lines SKOV3, ES2, HEY, and OVCAR were treated with 1 μM E2 for 48 h. The expression level of TRPC3 was detected by qRT-PCR. The experiment was repeated three times. The ovarian cancer cell line SKOV3 (**b**) was treated with 10 nM, 100 nM or 1 μM E2 for 48 h and HEY (**c**) treated with 1 μM E2 for 48 h. Western blot analysis was used to detect TRPC3 expression. β-Actin was used as a loading control

To further understand the molecular mechanism by which E2 promotes cell migration, we detected the expression of TRPC3 in different cells under E2 treatment conditions. E2 (1 μM) induced the upregulation of TRPC3 expression at the mRNA level (Fig. 4a). E2, especially 100 nM E2, also increased the expression of TRPC3 in SKOV3 and HEY cells at the protein level (Fig. 4b and c).

### Role of E2 in TRPC3-mediated Ca2+ uptake

**Fig. 5:**
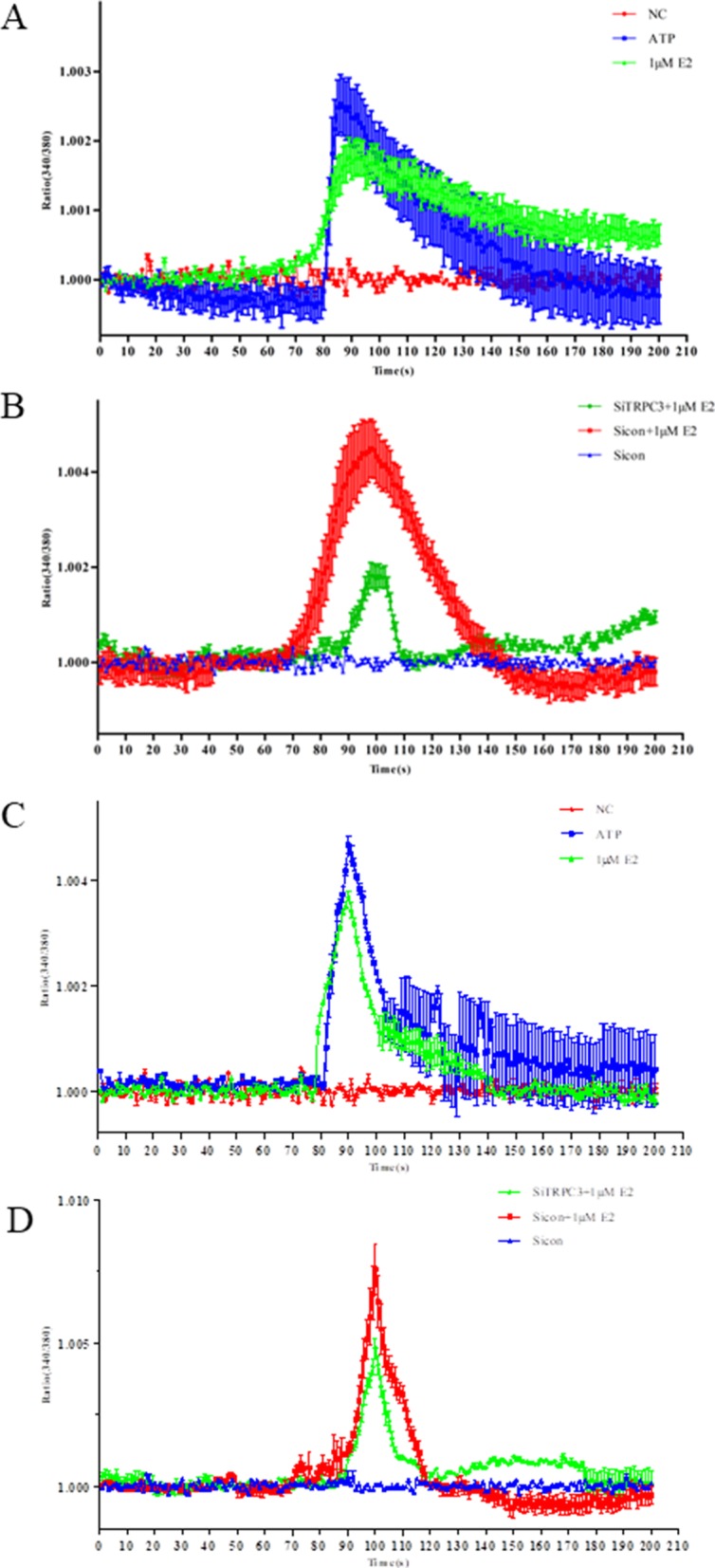
Knocking down TRPC3 expression blocked E2-associated effects on calcium influx. The effect of E2 on calcium influx is shown in (**a**, SKOV3 cells) and (**c**, HEY cells). ATP treatment was used as a positive control. A representative image of intracellular calcium influx ([Ca2^+^]_I_) is shown. The EOC cell line SKOV3 was used to analyze the effect of downregulating TRPC3 expression on [Ca2^+^]_I_ (**b**, SKOV3 cells) and (**d**, HEY cells). SiTRPC3 and Sicon were transfected into SKOV3 and HEY cells, which were then incubated on glass coverslips for 48 h. Then, 1 μM E2 was added to the medium at the start of detecting intracellular Ca2 + ., and then stained with the fluorescent dye Fluo-2 AM before observation. [Ca2^+^]_i_ was determined using the F340/380 ratio. The experiment was repeated three times

Previous studies have shown that TRPC3 acts as a Ca2 + −permeable channel in E2-responsive cells. To test whether E2 regulates TRPC3-mediated Ca2+ uptake, cells induced with 1 μM E2, ATP, and SiTRPC3 were analyzed using Ca2+ imaging. Treatment with 1 μM E2 facilitated intracellular calcium influx, which was consistent with the result of for ATP treatment in different cells (Fig. 5a and c). Intracellular calcium influx was lower when both SKOV3 cells and HEY cells were transfected with SiTRPC3 than transfected with Sicon when cells were treated with 1 μM E2 (Fig. 5b and d). The results suggested that specifically knocking down TRPC3 expression was associated with a block in rapid calcium influx. All results indicate that E2 influences intracellular calcium influx by influencing the expression of TRPC3.

## Discussion

EOC is the most lethal gynecological cancer and represents the seventh most commonly diagnosed cancer among women worldwide [21]. Approximately 75% of patients are diagnosed at an advanced stage because of the asymptomatic nature of EOC. Thus, it is urgent to understand the pathogenesis of EOC. E2 exhibits a broad spectrum of physiological functions ranging from regulation of the menstrual cycle and reproduction to modulation of bone density, brain function, and cholesterol mobilization [11, 12]. Notwithstanding the beneficial actions of endogenous E2, sustained exposure to exogenous E2 is an established risk factor for various cancers, especially those of the breast and endometrium [22]. It has been reported that an elevated E2 level is a risk factor for ovarian cancer, but the associated molecular mechanism is not clear. In the present work, we used E2 to stimulate different ovarian cancer cell lines to investigate the role of E2 in ovarian cancer.

To improve the treatment of EOC, disease pathogenesis is still being explored. Numerous studies have demonstrated the associations of E2 with the development and/or progression of various types of cancer, including cancers of the ovaries, lungs, and colon [13, 23]. Previous work revealed E2 as a risk factor for EOC [24, 25]. In the present study, we found that E2 increased the cell viability of different ovarian cancer cells, especially SKOV3 cells, and that it improved SKOV3 cell migration and proliferation in vitro (Figs. 1 and 3). The positive effects of E2 on the migration and proliferation of ovarian cancer cells suggest that E2 plays significant roles in the proliferation and migration of ovarian cancer. We also found that the expression of ER-α in different ovarian cancer cells was distinct. Taken together, these results demonstrate that the effect of E2 on EOC is mainly due to the role of EOC in promoting cancer cell migration and proliferation. However, the molecular mechanism by which E2 promotes cancer cell migration and proliferation is not clear.

Ca2^+^ signaling is believed to play a central role in the signaling cascades involved in tumorigenesis and neoplastic progression [26]. Inhibitors of Ca2^+^-dependent signaling suppress the proliferation of cancer cells in vitro and solid tumors in vivo [27, 28]. The canonical TRPCs are a subfamily that has been proposed to comprise protein tyrosine kinase- or G protein-coupled receptor-operated Ca2^+^ channels (ROCs) or internal Ca2^+^ store-operated channels (SOCs), which mediate the Ca2^+^ signaling pathway activated by many hormones and growth factors [15, 29]. TRPCs are ubiquitously distributed in the body and play essential roles in human physiology and pathophysiology. Several studies have demonstrated the expression of TRPCs in different types of cancer cells or cancer tissues [30–32]. Previous studies have shown that TRPC3 protein levels are significantly increased in human ovarian cancer specimens compared to normal ovarian tissue samples [20]. Consistent with this observation, in the present study, we found that TRPC3 expression was markedly increased in human ovarian cancer samples (Fig. 2). Our results and those of previous studies suggest that TRPC3 plays an important role in ovarian cancer. In this study, we found that the expression of TRPC3 increased after E2 stimulation. Intracellular calcium influx was lower when SKOV3 cells were transfected with SiTRPC3 than when SKOV3 cells were treated with 1 μM E2. These results indicated that E2 promotes the proliferation of ovarian cancer cells by upregulating the expression of TRPC3.

Because of the possibility of TRPC heteromultimerization, biological activities may involve more than one TRPC, which makes it challenging to identify the function of a single subtype [33]. Interestingly, we found that TRPC3 was involved in E2 regulation. Future work will address whether E2 influences other members of the TRPC family and the interrelations among the subtypes.

In summary, the results presented in this study demonstrated that the molecular mechanism of E2 involved promoting the proliferation of ovarian cancer cells. We demonstrated that E2 stimulated proliferation and migration by upregulating the expression of TRPC3, which enhanced intracellular calcium influx.

Additional future experiments might include overexpressing TRPC3 to observe the effects and experiments exploring the molecular mechanism by which estrogen regulates TRPC3 expression.

## Supplementary information

**Additional file 1: Figure S1.** Specific verification of TRPC3 used control peptide antigen. Control peptide antigen was used to demonstrate specificity of the band around 97 kDa. It showed that band around 97 kDa specifically represent TRPC3.

## Data Availability

The datasets used and/or analysed during the current study are available from the corresponding author on reasonable request.
