# Supplementary material for: Estrogen enhances the proliferation and migration of ovarian cancer cells by activating transient receptor potential channel C3
Source: J Ovarian Res. 2020 Feb 22;13:20. doi: 10.1186/s13048-020-00621-y (PMC7035653; doi:10.1186/s13048-020-00621-y)
Supplement: Supplementary file 1 — Additional file 1: Figure S1. Specific verification of TRPC3 used control peptide antigen. Control peptide antigen was used to demonstrate specificity of the band around 97 kDa. It showed that band around 97 kDa specifically represent TRPC3. [file 13048_2020_621_MOESM1_ESM.docx]

**
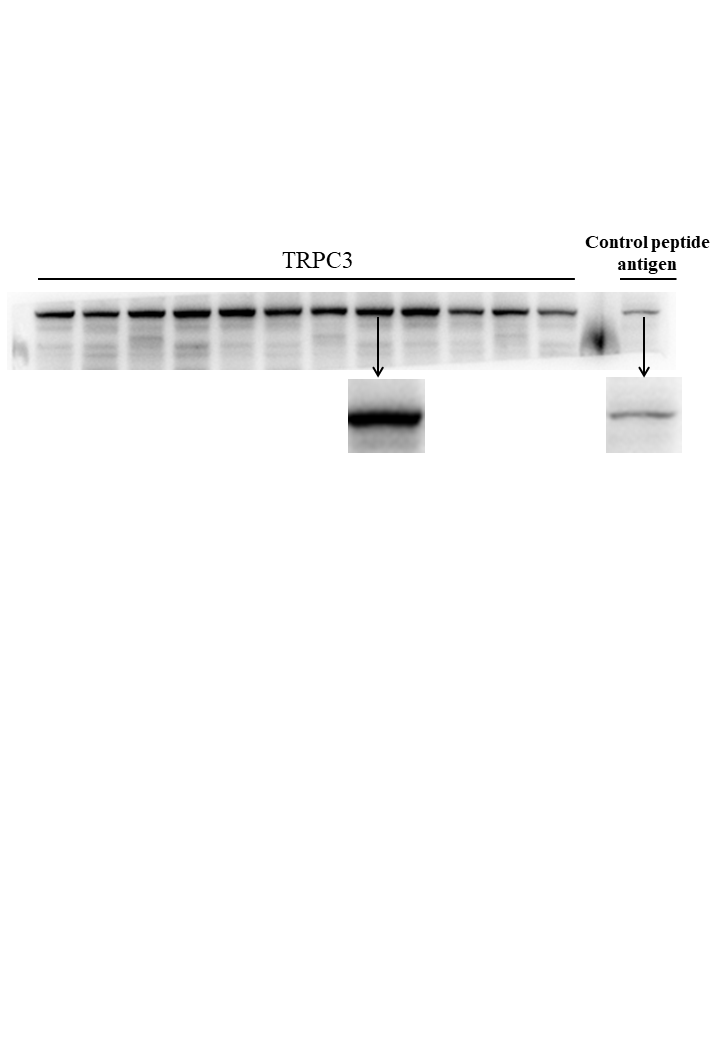
**

**Fig.S1. Specific verification of TRPC3 used** **control peptide antigen.**

Control peptide antigen was used to demonstrate specificity of the band around 97 kDa. It showed that band around 97 kDa specifically represent TRPC3.
